# The Role of Neurophysiology in Managing Patients with Chiari Malformations

**DOI:** 10.3390/jcm12206472

**Published:** 2023-10-11

**Authors:** Dulce Moncho, Maria A. Poca, Kimia Rahnama, M. Ángeles Sánchez Roldán, Daniela Santa-Cruz, Juan Sahuquillo

**Affiliations:** 1 Department of Clinical Neurophysiology, Vall d’Hebron University Hospital, Passeig Vall d’Hebron 119-129, 08035 Barcelona, Spain; kimia.rahnama@vallhebron.cat (K.R.); mariadelosangeles.sanchez@vallhebron.cat (M.Á.S.R.); danielaisabel.santacruz@vallhebron.cat (D.S.-C.); 2 Neurotraumatology and Neurosurgery Research Unit, Vall d’Hebron Institut de Recerca (VHIR), Vall d’Hebron Barcelona Hospital Campus, Passeig Vall d’Hebron 119-129, 08035 Barcelona, Spain; sahuquillo@neurotrauma.net; 3 Department of Neurosurgery, Vall d’Hebron University Hospital, Passeig Vall d’Hebron 119-129, 08035 Barcelona, Spain; 4 Department of Surgery, Universitat Autònoma de Barcelona, Bellaterra, 08193 Barcelona, Spain

**Keywords:** blink reflex, brainstem auditory evoked potentials, brainstem reflexes, Chiari type 1 malformation, electromyography, evoked potentials, intraoperative neurophysiological monitoring, motor evoked potentials, somatosensory evoked potentials, syringomyelia

## Abstract

Chiari malformation type 1 (CM1) includes various congenital anomalies that share ectopia of the cerebellar tonsils lower than the foramen magnum, in some cases associated with syringomyelia or hydrocephalus. CM1 can cause dysfunction of the brainstem, spinal cord, and cranial nerves. This functional alteration of the nervous system can be detected by various modalities of neurophysiological tests, such as brainstem auditory evoked potentials, somatosensory evoked potentials, motor evoked potentials, electromyography and nerve conduction studies of the cranial nerves and spinal roots, as well as brainstem reflexes. The main goal of this study is to review the findings of multimodal neurophysiological examinations in published studies of patients with CM1 and their indication in the diagnosis, treatment, and follow-up of these patients, as well as their utility in intraoperative monitoring.

## 1. Introduction

Chiari malformations (CMs) comprise a series of neurodevelopmental disorders characterized by a descent of the cerebellar tonsils through the foramen magnum (FM) [1,2,3,4,5]. CM type 1 (CM1) was first described by the Austrian pathologist Hans Chiari in two papers published in 1891 and 1895 [2,3,4]. Nevertheless, the term “Arnold-Chiari malformation” must still be introduced to search in PubMed or other databases for the literature on this abnormality. However, due to Arnold’s minor role in the original description, “Chiari malformation” is the most extended eponym [6]. In Chiari’s original description, four types of malformations were differentiated (CM1, CM2, CM3, and CM4). CM1 is traditionally defined as a tonsillar descent of 3–5 mm below the FM, quantified in a mid-sagittal section of magnetic resonance imaging (MRI). The choice of cut-off point for tonsillar descent (3 or 5 mm) is somewhat arbitrary and varies depending on the criteria of the different authors [7,8,9,10,11,12,13]. CM type 2 (CM2) is characterized by a descent of the structures of the brainstem or vermis below the FM and is always associated with spinal dysraphisms (spina bifida) in addition to tonsillar ectopia. Patients with CM2 also present a series of brain anomalies associated with spina bifida that are not observed in any other type of CM (gray matter heterotopia, polygyria, and descended tentorium, among others). CM type 3 (craniocervical encephalocele) and CM type 4 (cerebellar hypoplasia) [14] are severe malformations with a low incidence that most authors consider unrelated to CM1 and CM2. The recent International Consensus Conference recommended considering CM3 and CM4 as separate entities [15].

Other variants of CM1 have been described as minor or major forms of CM1. In 1998, Iskandar et al. coined the term “Chiari 0” (CM0) to describe five pediatric patients with syringomyelia, no tonsillar herniation, and a “tight” posterior fossa (PF), which improved after PF decompression. Subsequent studies by this and other groups have confirmed this entity and observed a significant volumetric reduction in the PF and alterations in cerebrospinal fluid (CSF) dynamics around the FM as the common etiopathogenic factor in CM0. Some authors suggested the term “tight cisterna magna” to define the same entity [16,17,18,19]. In 2004, Tubbs et al. coined the term “Chiari 1.5” to define those patients without spina bifida that presented, in addition to the tonsillar ectopia, a variable descent of the brainstem and displacement of the obex below the FM [13]. We found that once this variant was defined, a considerable percentage of patients in our series initially classified as CM1 actually corresponded to CM1.5 [19,20,21,22].

**Figure 1:**
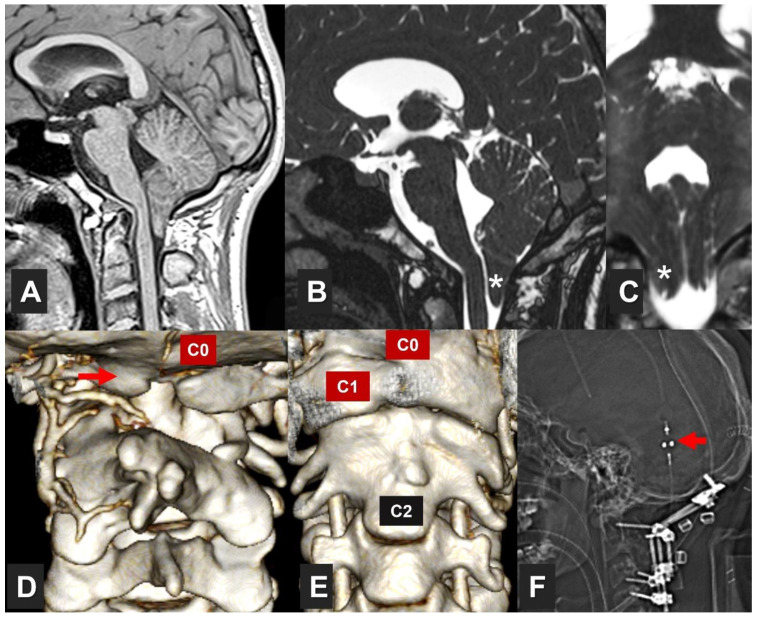
Patient with a complex craniovertebral junction (CVJ) malformation. This 17-year-old male with a VACTERL association (https://rarediseases.info.nih.gov/diseases/5443/vacterl-association) (accessed on 5 October 2023)—a non-random association of congenital disabilities that affects multiple parts of the body—was referred to our institution due to a Chiari malformation type 1 (CM1) detected as the patient presented recurrent episodes of severe occipital headaches. The neurological examination was normal. The patient had CM1 with an asymmetrical tonsillar descent 11 mm below the McRae line. (**A**) T1*weighted sagittal midline image; (**B**) T2*weighted sagittal, and (**C**) T2*weighted coronal slices; (**D**,**E**) three-dimensional CT reconstruction of the CVJ. White asterisks in (**B**,**C**) indicate the right cerebellar tonsil. The patient presented complete assimilation of the anterior and posterior C1 arches with an associated C2–C3 fusion and partial agenesis of the right C2 lamina. C0: posterior part of the foramen magnum. C1 labels the right lateral mass of the atlas. The red arrow in (**D**) shows the assimilated posterior C1 arch. Arrow in (**F**) indicates the valve (Polaris^®^ adjustable valve, Sophysa, Orsay, France) of a ventriculoperitoneal shunt implanted one month before because of hydrocephalus. This patient was treated with halo-vest stabilization for three weeks, followed by a one-step surgical procedure in which a posterior fossa reconstruction was conducted and instrumented occipito-C4 posterior fusion with bone-bank allograft (**F**).

An additional type, often not included in canonical classifications, is comprised of patients in which any type of CM is associated with different osseous malformations of the craniovertebral junction (CVJ). In a previous paper, we proposed the term ‘complex CVJ abnormalities’ when patients present tonsillar herniation and at least two of the following abnormalities: a significant retroflexed odontoid, a basilar impression (BI), platybasia, severe bone abnormalities in the C0–C2 complex, uni- or bilateral occipital condyle hypoplasia, atlantooccipital assimilation, or other abnormalities that condition an anterior compression of the cervical-medullary junction [19,23] (Figure 1). These patients need different clinical management and often require multiple surgical procedures (such as anterior approaches and occipito-cervical fusions) [24].

A common feature of CMs is that the cerebellar tonsillar descent causes compression of the neural structures and hinders CSF passage at the cervico–medullary joint, being able to alter brainstem and upper spinal cord function. This neural structures compression may generate dysfunctions in brainstem pathways, cranial nerve nuclei, or their exit from the brainstem, sleep-regulating regions, and cardiorespiratory centers [25]. The frequent association of syringomyelia with any form of CM can induce additional damage to the spinal cord and the spinal roots that emerge from the gray matter. These functional alterations of the nervous system can be detected by multimodal neurophysiological tests: brainstem auditory evoked potentials (BAEPs), somatosensory evoked potentials (SEPs), motor evoked potentials (MEPs), electromyography (EMG) of the cranial nerves/spinal roots, and brainstem reflexes.

Our paper aims to review the literature and summarize the findings reported in multimodal neurophysiological examinations in patients with CM0, CM1, and CM1.5, and their role in managing these patients, establishing indications for surgical treatment and follow-up. In addition, we will update their role in intraoperative neurophysiological monitoring (IONM). The role of neurophysiology in the diagnosis of sleep-related disorders, a frequent finding in CM1 [21,22,25], has deliberately been excluded from this review. We also excluded articles referring to cognitive evoked potentials.

## 2. Methods

**Figure 2:**
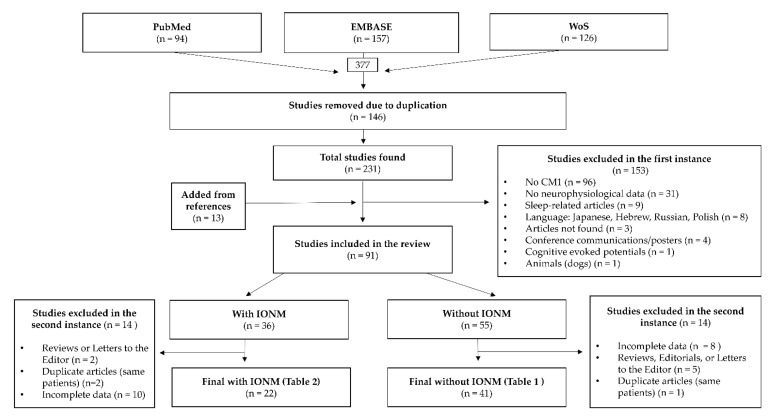
Flowchart. Diagram of the process used to select the articles for this review. CM1: Chiari malformation type 1. IONM: Intraoperative neurophysiological monitoring.

This study is an updated narrative review of neurophysiological studies (except for polysomnography and sleep disorders) published up until 30 August 2022. We searched the following databases: PubMed, EMBASE, and WoS. The search strategy used a combination of the keywords: “Arnold Chiari malformation,” “Chiari type 1 malformation*,” “Chiari type 1.5 malformation*,” “Type I Arnold Chiari malformation*,” “Chiari malformation type I with Syringomyelia,” “CM1 with Syringomyelia,” “Syringomyelia” and “evoked potentials,” “blink reflex*,” “brainstem auditory evoked potential*,” “brainstem reflex*,” “electromyography,” “motor evoked potential*,” and “intraoperative neuromonitor*.” The flowchart used is shown in Figure 2.

## 3. Exploring Brainstem and Spinal Cord Functionality

### 3.1. Brainstem Auditory Evoked Potentials (BAEPs)

**Figure 3:**
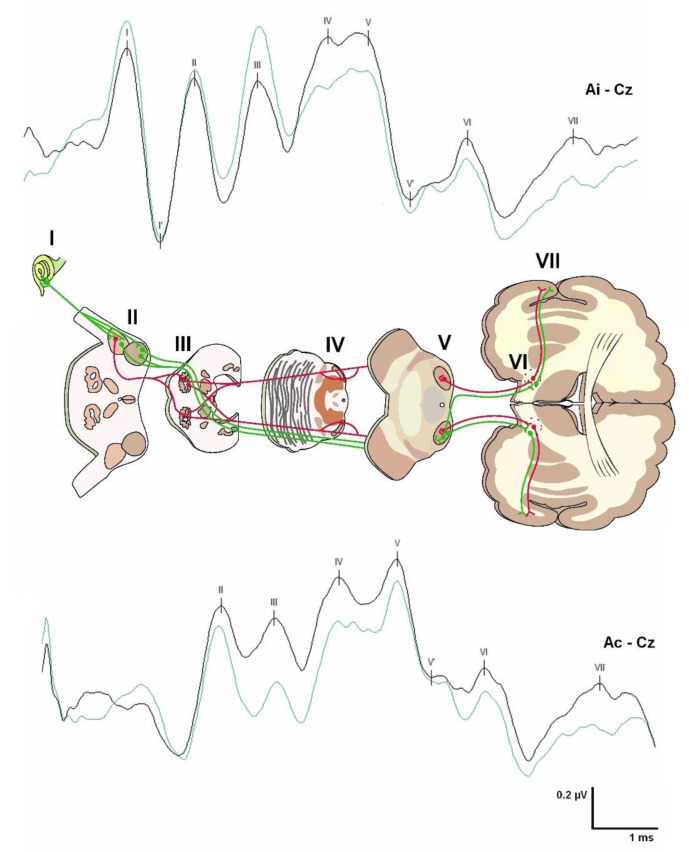
Example of normal brainstem auditory evoked potentials. The image summarizes the waves recorded after applying the acoustic stimulus with their respective neural generators. Wave I: organ of Corti of the cochlea (the distal portion of the auditory nerve). Wave II: cochlear nucleus (and the proximal intracranial portion of the auditory nerve). Wave III: superior olivary complex, trapezoid body. Wave IV: ventral nucleus of the lateral lemniscus. Wave V: inferior colliculus. Wave VI: medial geniculate nucleus of the thalamus. Wave VII: thalamocortical radiations and primary auditory cortex (Heschl’s) or Brodmann’s Area 41. The I’ and V’ cursors are used to measure the amplitude of waves I and V and thus be able to calculate the V/I amplitude ratio. Ai: ipsilateral earlobe. Ac: contralateral earlobe. (Modified from Moncho et al., with permission [23]).

BAEPs were first described in humans in 1970 [26]. They allow the recording of a series of five to seven positive electrical signals generated in response to sounds, each of which has a well-defined electrical generator [23,27] (Figure 3); because waves VI and VII are not constant in the healthy population, they are not routinely evaluated. BAEPs explore the functional integrity of a limited portion of the brainstem, both in the rostrocaudal direction—from the VIII cranial nerve entry at the pontomedullary junction to the upper part of the pons-midbrain—as well as in the transversal plane. The ventral part of the brainstem is not explored with BAEPs. It is widely accepted that alterations in waves I to III reflect the involvement of neural structures ipsilateral to the auditive stimulation. In contrast, abnormalities in the amplitude or latency of waves IV and V indicate contralateral structural impairment [27]. BAEPs can be abnormal in several processes: (1) peripheral auditory pathology (conductive or cochlear hearing loss); (2) primary anatomical modifications of the brainstem due to congenital pathologies (CM, Joubert syndrome, Dandy–Walker syndrome, etc.), or secondary (vascular, tumor, demyelinating, or degenerative); (3) anoxia and ischemia; and (4) intracranial hypertension with transtentorial herniation [23]. Due to the close relationship between the cranial nerves and the brainstem, BAEPs are routinely used in IONM and are important in surgical procedures in which the brainstem and cranial nerves are at risk [28].

### 3.2. Somatosensory Evoked Potentials (SEPs)

**Figure 4:**
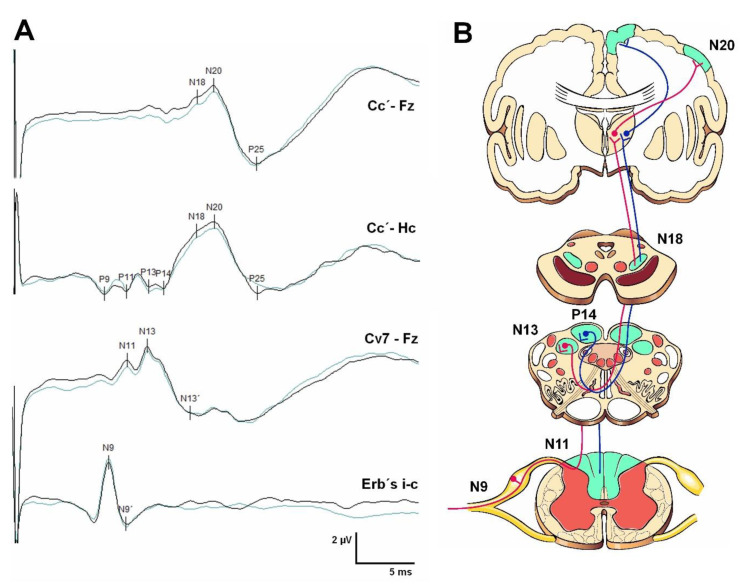
(**A**) Example of normal somatosensory evoked potentials. The image shows the responses recorded at different levels after stimulation of the median nerve in the wrist. (**B**) Anatomical drawing of its main neural generators. N9: brachial plexus. N11: entry of roots in dorsal horns and posterior columns. N13: high cervical and medullary structures (nuclei of the posterior columns). P14: most caudal part of the medial lemniscus. N18: medial lemniscus at the mesencephalic level. N20: primary somatosensory cortex (Brodmann areas 3, 1, 2). Cc’: contralateral parietal cortex. Hc: Contralateral shoulder. Cv7: the spinous process of the seventh cervical vertebra. (Modified from Moncho et al., with permission [23]).

SEPs explore the conduction of the electrical impulse through the lemniscal or dorsal column system (Figure 4), collecting the integrated responses obtained at different anatomical levels after applying a repetitive electrical stimulus on a peripheral sensory nerve or the sensory portion of a mixed nerve—usually the median nerve (MN) for the upper limbs and the posterior tibial nerve (PTN) for the lower limbs. In addition, other nerves, such as the cubital, trigeminal, saphenous, or pudendal, can be explored [29,30,31,32]. SEPs explore the somesthetic pathway from the peripheral nerve, preganglionic or postganglionic plexus, roots, spinal cord, brainstem, thalamus, and suprathalamic structures [23]. In anoxic or traumatic coma, they are useful for evaluating cortical and subcortical function. SEPs are now routinely used in the IONM of surgical procedures with a risk of damaging structures that generate and transmit the electrical signal [32]. In addition, ‘dorsal column mapping’ has also been introduced to guide the surgical team in procedures with a risk of spinal cord damage [33,34].

Cutaneous heat stimulations with a laser beam can selectively activate thermoalgesic A-delta and C nociceptors, leading to the generation of laser-evoked potentials (LEPs) in the cortex [35,36]. LEPs have been recognized as reliable neurophysiological tools for investigating neuropathic pain [37]. Pure spinothalamic lesions, such as syringomyelia, brainstem syndromes, or small fiber neuropathy, are characterized by normal SEPs but abnormal LEPs [36,38]. However, spinal injuries involving the cervical or lumbar dorsal horn can also alter or abolish components of classical SEPs, such as N13 or N22 [32,39]. Despite their potential utility, LEPs have not been widely adopted due to technical difficulties, the need for skilled personnel, and associated risks like skin burns and hyperpigmentation. Contact heat evoked potentials (CHEPs) have emerged as a safer alternative to LEPs and are easier to use but require patient cooperation and are unsuitable for IONM [40]. In recent research, Leandri et al. introduced a new noninvasive electrode that selectively stimulates nociceptive nerve-free endings, enabling the evaluation of disorders affecting the nociceptive pathway by enlarging the stimulated surface area [41].

### 3.3. Motor Evoked Potentials

The term “motor evoked potentials” refers to potentials recorded from muscle or nerve after stimulation of the primary motor areas in the CNS. MEPs are the responses obtained along the spinal cord, peripheral nerve, or muscle by stimulating the central motor paths (spinal cord or cerebral hemispheres) using either transcranial magnetic stimulation (TMS) or electrical stimulation. Electrical stimulation is utilized routinely for IONM, whereas magnetic stimulation is generally used for diagnostic studies in awake patients because of its better tolerance.

TMS is carried out using coils of different magnitudes and shapes. A magnetic field applied via the skull induces a stimulus in the underneath brain with minimal current affecting the skin and subcutaneous tissue. For lower extremity MEPs, the coil is placed in the Cz area; for the upper limb, it is just lateral to this reference. Per peripheral recordings, three MEP characteristics are employed: latency, amplitude, and threshold. MEP amplitude is highly variable but is generally considered abnormal if it is less than 20% of the compound muscle action potential (CMAP) amplitude obtained on peripheral neurography [42]. Central motor conduction time (CMCT) is the most commonly used parameter to identify CNS disorders [43] and is calculated by subtracting the latency of spinal MEPs from the latency of cortical MEPs [44]. TMS MEPs are used to study central demyelinating disease, motoneuron disease, epilepsy, movement disorders, ataxia, myelopathies, neuropathies, and radiculopathies [45].

In order to elicit an MEP by transcranial electric stimulation (TcMEP) for IONM, subcutaneous electrodes are positioned on the scalp in C1–C2 or C3–C4 (10–20 EEG International System). In general, a 50% reduction in amplitude from the average baseline value during surgery is considered significant and a warning sign of damage to the corticospinal tract. Similarly, corticobulbar motor evoked potentials (CoMEPs) elicit cranial nerve responses [46]. In addition to the two aforementioned techniques, which assess the integrity of the corticospinal tract, several mapping methods have been described for identifying the corticospinal tract by directly stimulating the exposed neural structures with a bipolar or monopolar electrode [47,48].

### 3.4. Electromyography and Nerve Conduction Studies

Practically all primary neuromuscular disorders cause changes in the electrical activity recorded in muscle fibers. These changes can best be explored employing needle electrodes introduced into the muscle to record free and voluntary electromyography (EMG). EMG is a technique that is basic for the diagnosis of motor unit disorders, involving anterior horn cells, peripheral nerves, neuromuscular junctions, and muscles. EMG complements nerve conduction studies (NCS) in localizing neuromuscular disorders [44,45]. Normal muscle fibers at rest show no spontaneous electrical activity except in the end-plate region. Voluntary muscle activity is produced by the lower motor neurons and their corresponding innervated muscle fibers that form the ‘motor unit potentials ‘(MUPs). The extent and distribution of the EMG patterns allow us to define the type and severity of the disease, its stage, and the anatomical location of the lesion [44].

EMG is also used in IONM via continuous free-run and stimulated EMG. Free-run EMG records spontaneous muscle activity in real time to detect surgically induced mechanical irritation of the peripheral and cranial nerves before irreversible damage may occur. Stimulated EMG consists of electrically stimulating the peripheral motor nerves or roots registering in the corresponding muscles and can be used to localize peripheral or cranial nerves that are difficult to distinguish from tumors, fibers, or fatty tissues [49].

### 3.5. Brainstem Reflexes

**Figure 5:**
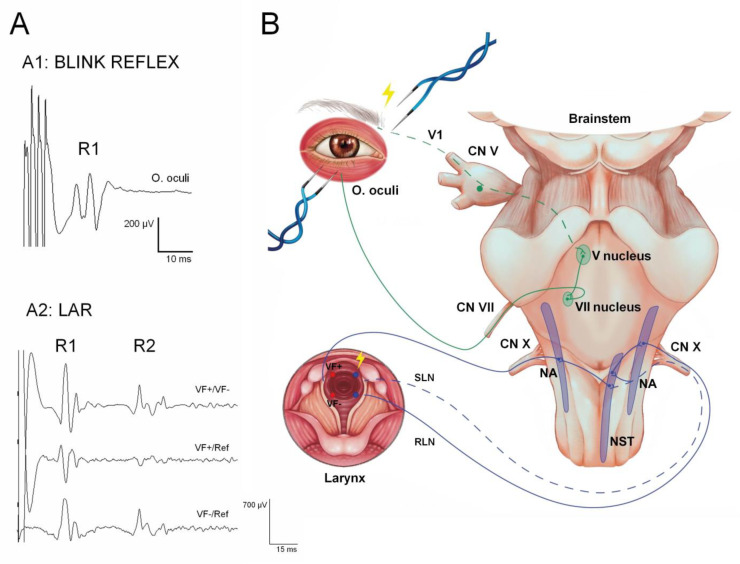
Blink reflex and laryngeal adductor reflex. Panel (**A**) shows exemplary traces of the R1 response of the blink reflex (BR) (**A1**) and the R1 and R2 components of the laryngeal adductor reflex (LAR) (**A2**) intraoperatively recorded under general anesthesia. Panel (**B**) illustration represents the setup used to stimulate and record the BR (top scheme, green lines) and the LAR (bottom scheme, purple lines), including a schematic representation of the arc reflex pathways (please note that discontinued lines are used for afferent fibers and continued lines for efferent pathways), and the brainstem structures involved. V1 is the first branch of the cranial nerve V; CN: cranial nerve; NA: ambiguous nucleus; NST: nucleus of the solitary tract; RLN: recurrent laryngeal nerve; SLN: superior laryngeal nerve; VF: vocal fold.

Blink reflex. The blink reflex (BR) is the neural response obtained in the orbicularis oculi muscle after stimulating the trigeminal nerve with electrical, mechanical, or other stimuli. The BR is the most widely used brainstem reflex in clinical practice. The trigeminal BR is mediated by the first division of the trigeminal nerve (afferent branch) and facial nerve (efferent branch). In normal individuals, two responses (R1 and R2) are obtained when recording from the ipsilateral muscles to the stimulated nerve, and a single (R2) response is obtained from the contralateral muscles [50]. R1 is constituted by the oligosynaptic reflex arc, which includes trigeminal afferents, brainstem connections between the sensory part of the trigeminal nucleus, the motor nucleus of the facial nerve, the facial nerve proper, and the orbicularis oculi muscle (Figure 5). The R2 component has polysynaptic connections within the brainstem but has the same afferent/efferent pathways as R1. BR is useful in the study of trigeminal and facial nerve lesions, peripheral neuropathy, posterior fossa lesions, multiple sclerosis, and extrapyramidal diseases [51]. Under general anesthesia in the operating room, only the R1 response can be recorded [52,53,54,55].

Trigeminal–hypoglossal reflex. Brainstem trigeminal–hypoglossal reflexes (THRs), also known as jaw-tongue reflexes, coordinate the tongue’s position in the mouth during chewing, swallowing, vocalization, and breathing. Recently, a novel methodology for obtaining jaw-opening THR of the brainstem under general anesthesia has been described. This technique could be helpful for the intraoperative monitoring of surgeries involving the trigeminal, hypoglossal nerves, and lower brainstem lesions; however, further studies are still required to validate it [56,57,58,59,60].

H reflex in the masseter muscle. The H reflex in the masseter muscle is a monosynaptic trigeminal–trigeminal reflex transmitted through the mesencephalic nucleus of the trigeminal nerve. It reflects conduction through the midbrain and the median pons [61,62,63]. It can be helpful in studying patients with brainstem or trigeminal lesions, and can be obtained under general anesthesia, thus representing a new method for intraoperative monitoring, especially for lesions involving the midbrain and pons. Together with the BR, the H reflex is a straightforward method for intraoperative monitoring of brainstem reflex circuits and the functional integrity of the trigeminal and facial nerves. Nevertheless, even more studies are necessary to demonstrate their usefulness [64].

Laryngeal adductor reflex. Recording of the laryngeal adductor reflex (LAR), a brainstem reflex entirely governed by the vagus nerve, has been introduced to monitor the vagus and laryngeal nerves in thyroid surgery [65,66]. Preservation of the reflex prevents potentially harmful substances from entering the tracheobronchial tree by activating the adductor muscles of the larynx to close both vocal folds [67,68]. Recently, a study was published using this reflex in surgeries around the brainstem to monitor cranial nerve X and related intrinsic brainstem pathways (Figure 5), showing that IONM with continuous LAR monitoring, together with vocal-CoMEPs, may be useful in reducing swallowing and laryngeal complications, such as aspiration and pneumonia, enhancing safety in resecting complex PF lesions [69,70].

## 4. Neurophysiological Limitations in the Developing Central Nervous System

Neurophysiological test references change during the maturational process of the CNS and, therefore, exhibit some peculiarities according to the patient’s age. In full-term newborns, the most reproducible waves in BAEPs are I, III, and V [71]. However, unlike adults, it is normal for wave I to have a greater amplitude than complex IV/V. The myelin maturation process shortens the latency of the successive waves and the central conduction time (CCT or I-V interval). It has also been demonstrated that the auditory pathway responsible for generating the first and last components of BAEP responses matures at a different rate [72]. In a normal subject, wave I latency reaches the adult value around six months of life, while wave V does so by one year of life [71].

A similar process occurs in SEPs; the maturation of peripheral segments of the sensory pathway progresses more rapidly than that of central segments. N9 latency—brachial plexus potential—shortens during the first year of life and subsequently increases as the patient’s size increases. The N9–N11 interval—conduction between the brachial plexus and the dorsal column—decreases with age. In contrast, the N13–N20 (CCT) presents a rapid shortening during the first year of life, reaching adult characteristics at four years of age [73].

Independent of the changes described, the motor stimulation threshold is higher in children than in adults under 18 years of age [74]. CMCT is a dependent value of CNS maturation but has different times. For example, with relaxation (resting motor threshold), children reach adult values approximately at the age of ten; however, with facilitation (active motor threshold), they reach normal values at two years of age [74,75]. Also, MEPs could be obtained at a resting motor threshold in the upper limb after the first year of life and in the lower limb after four years. Maturation also affects the duration, morphology, and amplitude of MEPs.

When evaluating peripheral nerves in children, the myelination process also affects parameters commonly used for assessing NCS [76]. For instance, conduction velocity increases in proportion to nerve fiber diameters and the distance between the nodes of Ranvier, achieving faster motor and sensitive velocities with growth; distal latencies and amplitudes also change. For example, the CMAP increases threefold in the upper limbs and twofold in the lower limbs [76].

The LAR in children is vital for airway protection. In humans, the larynx descends from the neck at 4–6 months. This descent coincides with the transition from obligatory nasal breathing in infants to facultative nasal or oral breathing in adults. It is possible that alterations in this transition are related to sudden infant death syndrome, whose frequency is maximum at the same age at which laryngeal descent is completed (4 to 6 months) [77,78]. In theory, as of six months of life, we should record the LAR (either in the clinic or the operating theatre), although it may be necessary to do so with hook wire electrodes if there is no age-appropriate endotracheal tube [79,80].

A discussion of the changes from children to adults is beyond the scope of this paper. However, it is essential to bear in mind that neurophysiological tests have some peculiarities according to age, basically due to myelin maturation, which must be considered when evaluating children.

## 5. Follow-Up Neurophysiological Studies in Patients with CM1

There are many publications on the follow-up of CM1 patients; however, practically half of them refer to case reports or series with fewer than ten patients (Table 1). Here, we summarize the most relevant information.

### 5.1. Brainstem Auditory Evoked Potentials

To our knowledge, the first paper was published in 1983 by Stone et al. [81], describing BAEP findings in a 16-year-old boy with CM2 and symptoms suggestive of brainstem involvement and neuroimaging revealing brainstem compression. The authors diagnosed CM2; however, as the reported patient did not have any visible spinal lesion, we believe that CM1.5 was the most likely diagnosis [13]. BAEPs were abnormal bilaterally, with I-V interpeak latency (IPL) prolongations and no visible wave III identified upon stimulation of the right ear. The patient underwent bilateral posterior fossa decompression (PFD) and cervical laminectomy. Six months later, he presented neurological improvement and bilateral normalization of his BAEPs. A few authors have also described case reports with retrocochlear findings in the BAEPs of CM1 patients, in both adults [82,83] and children [84,85] (Table 1).

In 1999, Hort-Legrand and Emery [86] reported 79 patients with syringomyelia, of which 48 cases were associated with CM1. BAEPs were abnormal in 13 of the 59 patients studied, and the most frequent finding was prolongation of the I-V IPL, more often unilaterally. Another study on syringomyelia and CM1 [87] found that BAEP abnormalities had a better correlation with clinical and radiological findings than SEPs.

In 2006, Henriques Filho and Pratesi used BAEPs to study 75 CM1 and CM2 patients [88]. Abnormalities were detected in most cases (71%); however, more were observed in CM2 patients. According to these authors, these findings reinforce the concept that pontine alterations are a consequence of brainstem dysgenesis in CM2. They also observed that patients with CM1 preferentially had peripheral alterations—cochlea or cochlear nerve close to the cochlea—with less in the area located between the cochlear nerve and the pontomedullary junction. This study concludes that the assessment of the V/I amplitude ratio, together with other abnormalities, allows the identification of a more significant number of BAEP alterations in patients with CM1 and is a valuable tool for the diagnosis. Vidmer et al. [89], in 2011, described BAEP findings in a CM1 nine-year-old girl, in whom the abnormalities were found at the peripheral or cochlear level, supporting the results of Henriques Filho and Pratesi [88].

In studies carried out by our group―the latest including 200 patients―we found that BAEPs were altered in 38.5% of patients with CM1 [19,20]. The most frequent finding was increased I–V interval and wave V latency (31%). The logistic regression model showed that age, the degree of tonsillar ectopia, and the clinical detection of lower cranial nerve involvement had statistical significance in predicting pathological BAEPs. This result suggests that a more awful alteration of the brainstem induces more BAEP abnormalities. Consequently, patients with a greater degree of tonsillar ectopia or lower cranial nerve involvement had a greater rate of pathological BAEPs.

Di Stefano et al. studied BAEPs and MRI in three groups of patients: with intracranial hypotension (*n* = 18), CM1 without intracranial hypotension (*n* = 18), and sensorineural hearing loss (*n* = 20) vs. controls (*n* = 52). In the CM1 group, they found abnormal BAEPs in 33% of patients, the main finding being a prolonged latency of wave V and I-III and III-V intervals [90], with comparable results to those obtained by our group [19,20].

### 5.2. Somatosensory Evoked Potentials

In 1986, Anderson et al. [91] described the SEPs of nine syringomyelia patients, eight with associated CM1. The most frequent findings were unilateral or bilateral decrease or absence of the cervical potential. In all but one patient with CM1 and syringomyelia, an increased or asymmetric CCT was found; in 1988, Forcadas et al. reported similar results [92]. In 1990, Jabbari et al. published a study with SEPs from the MN and PTN in 22 patients with syringomyelia, four with CM. Three of the four patients with CM1 and syringomyelia had normal SEPs [93]. Restuccia and Mauguière [39] studied the MN SEPs of 24 patients with syringomyelia, 16 associated with CM1. They observed several anomalies, the most common being an abnormal or absent N13 wave in 83% of patients with cervical syringomyelia. When syringomyelia became associated with CM, these authors, in contrast to Jabbari et al. [93], observed an increase in the P14-N20 interval; Morioka et al. reported similar results [94]. Several case reports and small series with SEP investigations in CM1 patients show abnormal SEPs at a central level, with an absence of cortical responses and an increased latency of cortical potentials or CCT [89,95,96,97].

Moncho et al. found that SEPs were altered in 43.5% of CM1 patients. The most common finding in MN SEPs was an increase in N13-N20 IPL, while for PTN SEPs, it was an increase in N22-P37 IPL, sometimes associated with an abnormal cervical response. In a logistic regression model, only age and tonsillar herniation degree showed statistical significance for predicting abnormal SEPs. These results indicated a higher chance of obtaining pathological SEPs in older patients with greater tonsillar descent [19,20]; recently, Guvenc et al. described similar findings [98].

Some studies specify the relation between idiopathic scoliosis and CM1, in which abnormal SEPs can be due to tonsillar ectopia or syringomyelia rather than spinal deformity [95,99]. Cheng et al. found a relationship between tonsillar ectopia and SEP alterations when studying 164 patients with idiopathic scoliosis, 12 of whom had associated CM1 [99]. Utzig et al. reported the case of a 15-year-old girl complaining of intense headache, recurrent left-sided paresthesia, and progressing scoliosis, who had CM1 with cervical syringomyelia. Ulnar and PTN SEPs showed an absence of cortical responses that improved after surgical treatment with PFD, C1 laminectomy, and partial removal of the cerebellar tonsils with the reappearance of cortical responses of acceptable latency but with reduced amplitude for the left extremities [95].

The somatosensory tract in CM1 can also be evaluated by trigeminal SEPs from V3, which can present prolonged latency. Hort-Legrand and Emery found that trigeminal SEPs were much more sensitive than BAEPs, being altered when patients had bulbar symptoms [86].

Berciano et al. reported a CM1 patient who presented with sudden lancinating episodes of left cervicobrachial pain caused by cough attacks, related to cervico-dorsal syringomyelia spreading into the left posterolateral quadrant in axial slices running through vertebral levels C7 and D1. Pre- and postoperative upper limb dermatomal SEPs (dSEPs) elicited by stimulation at levels C6, C7, and C8 showed N20 attenuation only after stimulation of the left fifth finger (left C8 level). Given the preservation of peripheral sensory nerve action potentials (SNAPs) in both upper limbs, such a selective dSEP attenuation is probably caused by extension of the paracentral posterior horn cavity into the C8 posterior entry zone. Under such circumstances, dSEP is a useful method of evaluating this radicular semiology [100]. In 2015, Awai and Curt reported seven patients with syringomyelia showing differences in dSEP and CHEP affectations. Dermatomal CHEPs were abnormal in all patients in at least one dermatome [101].

### 5.3. Motor Evoked Potentials

Few authors have described TMS MEP alterations in patients with CM1, most of them with syringomyelia. The most frequent findings were increased CMTCs [86,102,103,104].

Referring to pre- and post-surgical MEP evaluations, Cristante et al. [105] described the MEP findings in eight CM1 patients. Preoperative TMS MEPs showed that functionally impaired muscles exhibited neurophysiologic abnormalities, and even one patient without any motor deficits had abnormal MEPs. Interestingly, the postoperative functional motor recovery of five of the eight patients―mostly partial―was not reflected by a similar improvement in MEPs.

### 5.4. NCS, EMG, and Other Peripheral Nerve Studies

In 1992, Gerard et al. described the case of a five-year-old girl―with CM1 and BI―admitted due to insufficiency of the soft palate [106]. EMG activity was recorded bilaterally in the levator palatini and anterior faucial pillars, showing ample biphasic or polyphasic action potentials at rest (probably spontaneous activity). When the child cried, the frequency of these potentials increased poorly, and recruitment was impaired. Clinical examination and EMG findings led to a suspicion of denervation of the IX, X, and XI cranial nerves.

CM1 should be a differential diagnosis in patients with adult dysphagia onset requiring MRI examination, even when presenting with “typical” lower motor neuron signs in bulbar muscles. SEPs, NCS, and EMG can help diagnose these cases [107].

In patients with CM1 and associated syringomyelia, NCS and EMG are useful in detecting anterior horn affectation caused by the syringomyelia. This syndrome characteristically presents with spontaneous activity while performing needle EMG if acute or denervating activity occurs, with large, polyphasic (neurogenic) MUPs, reduced voluntary recruitment, and normal sensory nerve conductions [108,109,110,111,112,113]. Sometimes, this can be misdiagnosed as focal mononeuropathy if only motor NCSs are performed since a common finding is a small amplitude CMAP [108,109,110,112,113], although just one group reported unaffected CMAPs [111]. Another study found a confounding ulnar neuropathy with a tricky focal slowing at the elbow in a patient with cervical syringomyelia at C7-D1. A few studies reported a clinical foot drop syndrome in children, with neurogenic findings in most muscles that were dependent on the common peroneal nerve but also the PTN, and with normal sensory evaluation. The MRI confirmed the presence of holocord syrinx [110,111,112,113,114]. Therefore, it is essential to keep in mind that foot drop is a common problem that can present, and even though the most frequent cause is peroneal neuropathy, other causes include anterior horn cell disease, lumbar plexopathies, L5 radiculopathy, partial sciatic neuropathy, and, rarely, parasagittal lesion.

Painful cutaneous nerve stimulation can suppress EMG activity in voluntarily contracting muscle [115,116,117,118,119]. This phenomenon is designated the “cutaneous silent period” (CSP). In 1996, Kaneko et al. [120] studied five male patients (mean age 18 years) with cervical syringomyelia using CSPs, CMAPs, F waves, MEPs, and SEPs of upper limbs. MEP latency and amplitude were within normal limits in all patients. Three of the five patients presented absent or reduced cervical N13 potentials from SEPs of the upper limbs with preserved cortical responses. In the symptomatic hand, all patients exhibited reduced CSPs, constituting the only abnormal finding in two subjects. The laterality of the abnormal CSPs and the reduced N13 potentials of the SEPs were consistent with the site of the syrinx on MRI. Previous reports demonstrated that absent or reduced cervical N13 potentials with preserved scalp responses of upper limb SEPs are characteristic of early cervical syringomyelia [39,91,92]. Kanebo et al. further demonstrated that the absence of CSPs with normal conduction in large, myelinated fibers that form motor and sensory pathways is characteristic of patients with cervical syringomyelia. In addition, the CSP decrease was more sensitive to syringomyelic changes than abnormal cervical N13 potentials were.

### 5.5. Brainstem Reflexes

We only detected two reports regarding CM1 follow-up with brainstem reflexes, specifically, the blink reflex. Amoiridis et al. [121] reported a case of a 25-year-old man with CM1 and holocord syrinx. They performed the BR that showed Rl absent bilaterally and R2 latency with left-side stimulation prolonged on both sides, even though the patient did not present any anomaly in clinical cranial nerve exploration. Jacome, in 2001, described four CM1 patients presenting with blepharoclonus, one with altered R1, two with altered R2, and one with normal BR. Also, facial EMG showed complex repetitive discharges of the right mentalis muscle in one patient [122].

**Table 1:** Published neurophysiological follow-up studies in patients with Chiari 1 malformation.

Author	Year	N	Age/Sex	SYR	SCOL	BAEPs	SEPs	MEPs	EMG/NCS	Brainstem Reflexes
Cocito et al. [104]	2022	100 Symptomatic CM1 = 34 CM1 + SYR = 53 SYR = 13	23–75-y-o 25♂/75♀	66	―	―	―	TMS of phrenic nerve: - 48%: ABN C5-MEP. - 20%: absence or delay of Cz-MEP Alterations of the Cz-MEP and C5-MEP were prevalent in patients with cervical SYR/syringobulbia, most associated with CM1	―	―
Di Stefano et al. [90]	2019	Comparison between groups: IH = 18 CM1 = 18 SNHL = 20 Normal controls = 52	CM1 49 ± 11-y-o 12♂/6♀	―	―	Wave V, I–III, and III–V IPL were higher in CM1 than in controls	―	―	―	―
Guvenc et al. [98]	2019	T = 27	15–62-y-o 7♂/20♀	―	―	―	ABN SEP in 22.2% (PTN > MN). - Significant correlation between CSF flow disturbance, the degree of TE (*p* = 0.038), and the presence of SEP abnormality (*p* = 0.016). - CSF flow disturbance and SEP abnormality are more frequently seen in patients with platybasia	―	―	―
Jayamanne et al. [111]	2018	T = 1 Presenting as left foot drop	6-y-o ♀	YES (holocord SYR)	―	―	―	―	NCS: CPN, PTN, and sural nerve were normal. EMG at left TA revealed fibrillations and scanty MUP. EMG of left medial gastrocnemius and right TA were normal	―
Stancanelli et al. [102]	2018	T = 1 Presenting as excessive sweating on all the left side of the body	22-y-o ♂	―	―	Normal	Normal	Asymmetric response with increased CMCT on the right upper and lower limbs	Sudoscan test: Asymmetric sweating with a higher electrochemical skin conductance on the left hand and foot	―
Moncho et al. [19]	2017	200	15–70-y-o 58♂/142♀ CM0 = 14 CM1 = 137 CM1.5 = 49	YES (96)	―	Only age, the degree of TE, and lower cranial nerve dysfunction had a statistically significant influence in predicting ABN BAEPs	Only age and the degree of TE were statistically significant at predicting ABN SEPs. BAEPs and SEPs play an essential role in clinically asymptomatic/oligosymptomatic patients	―	―	―
Akakin et al. [96]	2015	T = 1	34-y-o ♂	Large SYR from just below FM to T5 vertebral body. The spinal cord was thinned at these levels	―	―	Preoperatively SEPs were ABN: Increased N13-20 IPL of the MN SEP and reduced cortical AMP of the PTN SEP. After syringo-subarachnoid-peritoneal shunt insertion using a conventional lumbo-peritoneal shunt and a T-tub his SEP test turned to normal	―	―	―
Awai and Curt [101]	2015	T = 7 SYR (1 CM1)	32–53-y-o 6♂/1♀ MC1: 32-y-o ♂	YES	―	―	Differently affected dSEPs and dCHEPs. dCHEPs in at least one dermatome were ABN in all patients	―	All patients had normal NCS and MEPs of the upper limbs	―
Moncho et al. [20]	2015	50	16–67-y-o 11 ♂/39♀	YES (20 patients)	―	Altered in 52%. The most common finding was an increased I–V IPL and LAT of wave V (48%). A greater TE was observed in patients with pathological BAEPs compared to patients with normal BAEPs (not statistically significant, possible type II error)	Altered by 50%. The most frequent alteration of MN SEP was increased N13-N20 IPL; the most frequent alteration of PTN SEP was an increased N22-P37 IPL, sometimes associated with an alteration of the cervical potential. A greater TE was observed in patients with altered PTN SEPs, and MN SEPs compared to patients with normal SEPs (not statistically significant, possible type II error)	―	―	―
Isik et al. [87]	2013	T = 44 SYR CM1 = 32	14–71-y-o 24♂/20♀	YES	―	Only pathological in 31.2% of patients with CM1. In 90% of cases improvement was seen and correlated with neurological and radiological improvement. This series suggested that BAEPs were more correlated with clinical and radiological findings than SEPs were	MN/PTN SEPs were ABN in 54.2% of patients (exact number of patients not known since the figures do not match). SEPs were not always correlated with the clinical findings	―	―	―
Panda and Kaur [114]	2013	T = 1 Rapidly progressive right foot drop	16-y-o ♂	YES (holocord SYR)	―	―	―	―	NCS: CPN normal BIL with absent bilateral F waves. PTN, sural, superficial peroneal, and saphenous nerve were normal. EMG: fibrillation potentials and positive sharp waves in the right TA, peroneus longus, medial gastrocnemius, gluteus medius, and lumbar paraspinal muscles, confirming the lesion to be proximal (lumbar roots or anterior horn cell)	―
Vidmer et al. [89]	2011	T = 66 (MC1 and 2) 1 MC1	3 months-60-y-o MC1: 9-y-o ♀	―	―	Peripheral or cochlear alteration in a single pediatric patient with CM1	SEP of MN ABN with increased LAT N20 and CCT UNIL Normal PTN SEP BIL	―	―	―
Mc Millan et al. [113]	2011	T = 2 Abrupt onset UNIL foot drop	5 and 4.5-y-o 2♀	YES	―	―	Case (1) Not included or provided. Case (2) MN SEPs revealed prolonged cervical and cortical responses. PTN SEPs responses were poorly formed with normal latencies	―	NCS: Case (1) CPN showed low motor AMP. EMG: fibrillation potentials and positive sharp waves confirming a proximal lesion. Case (2) EMG of the right TA revealed active denervation	―
Saifudheen et al. [112]	2011	T = 1 Rapidly progressive BIL foot drop	14-y-o ♀	YES (holocord SYR)	―	―	―	―	NCS: Low AMP CMAP and normal LAT and velocity for both peroneal and left ulnar nerves. The F wave was normal. Sensory median, ulnar, and sural nerves were normal. EMG: On both sides, chronic neurogenic changes in the first dorsal interossei, TA, and medial gastrocnemius muscles	―
Berciano et al. [100]	2007	T = 1 Lancinating left cervico-brachial pain provoked by coughing fits	54-y-o ♀	Cervico-dorsal SYR extending into the left posterolateral quadrant in axial sections passing through C7 and D1 vertebral levels	―	―	dSEPs left side: N20 of the C8 dermatome severely attenuated, both pre- and postoperatively. All other left-side dermatomes and right-hand dSEPs were normal Persistence of segmental hypoalgesia and altered SEPs despite the disappearance of SYR on MRI after PFD	―	Bilateral motor and sensory conduction parameters of MN and UN, including F responses, were normal	―
Henriques Filho and Pratesi [88]	2006	T = 75 MC1 = 27 MC2 = 48	27 patients = 19–70-y-o 48 patients = 2–16-y-o	―	―	In order of frequency: 1. Alteration of wave I or cochlear level (“segment 1”); 2. Alteration I-III or “between the acoustic nerve near the cochlea and the pontomedullary junction” (“segment 2”). -Two patients with an abnormality in the AMP V/I ratio	―	―	―	―
Brookler [82]	2005	T = 1 Dizziness	63-y-o ♀	―	―	Delay of wave V and III–V BIL, with normal audiology	―	―	―	―
Utzig et al. [95]	2003	T = 1 Headache, paresthesia, and SCOL	15-y-o ♀	YES	YES	―	Left UN/PTN SEPs: Absence of cortical responses. After SUR: Cortical responses of reduced AMP for left extremities	―	―	―
Hausmann et al. [123]	2003	T = 100 MC1 = 1	15.3 ± 2.2-y-o 20♂/80♀	3	100	―	Normality in PTN SEPs in the patient with MC1	―	―	―
Muhn et al. [110]	2002	T = 1 Rapidly progressive UNIL leg weakness	5-y-o ♂	YES	―	―	―	―	NCS showed a low-AMP CMAP for the left peroneal and normal sural nerve. EMG of the left TA, tibialis posterior, and medial gastrocnemius muscles showed fibrillation potentials at rest and reduced voluntary recruitment action potentials. Eight weeks after PFD, patient showed improvement in leg strength with a co-temporal resolution of the previously observed fibrillation potentials	―
Paulig and Prosiegel [107]	2002	T = 1 Progressive dysphagia for a year	78-y-o ♀	―	―	―	Normal	―	-NRL examination: BIL paresis and atrophy of the tongue showed diffuse fibrillations. Further neurological examination was normal. -NCS and EMG of various muscles of the upper and lower limbs were normal	―
Bagnato et al. [108]	2001	T = 1 Presenting as spinal myoclonus	48-y-o ♂	YES	―	―	Left MN SEPs revealed normal P14 and N20, while the N13, obtained after glottis reference, was nearly abolished	―	EMG: (1) Chronic partial denervation in C8/D1 muscles; (2) rhythmic contractions in FDI and ABP muscles (spinal myoclonus); and (3) peripheral silent period after supramaximal electrical stimulation of UN at FDI muscle	―
Jacome [122]	2001	T = 4 CM1 presented with blepharoclonus	17–52-y-o 1♂/3♀	1	―	―	―	―	Facial EMG: complex repetitive discharges of the right mentalis muscle in one patient	Blink reflexes were ABN in 3 patients
Scelsa [109]	2000	T = 1 Presenting as ulnar neuropathy at the elbow	24-y-o ♀	YES	YES	―	―	―	NCS: Marked AMP reduction of the left ulnar CMAP without focal slowing or conduction block across the elbow. EMG: Fibrillations and high AMP MUP in left arm muscles innervated by C7-D1 spinal segments and the median and ulnar nerves	―
Cheng et al. [99]	1999	T = 164 MC1 = 12	10–20-y-o (m₁ = 14.2) (m₂ = 13.6) 22♂/142♀	6	164	―	MN and PTN SEPs. Association between TE and SEP dysfunction (*p* < 0.001; c.c 0.672 Spearman). No differences in the degree of TE in patients with normal SEPs and those with ABN SEPs (*p* = 0.864; Mann–Whitney)	―	―	―
Hort-Legrand and Emery [86]	1999	T = 79 SYR -Foraminal (64) -Meningitis (5) -Trauma (15) CM1 = 48 (CVJM = 11 BI = 7)	SYR foraminal 16–71-y-o 27♂/22♀	YES	11	ABN in 13 of the 59 patients studied (total cohort), 6 with CVJM. The more frequent finding was I-V IPL PROL UNIL, less frequently BIL. (They did not specify how many patients with CM1 underwent BAEP or what the results were in this subgroup)	MN and PTN SEPs in all 79 patients. Abnormality (PTN or MN) was noted in 77 of the 79 patients. The most frequent findings for MN SEPs were altered cervical N13 response, an anomaly of the P14-N20 interval, or altered cortical response. The most frequent findings of PTN SEP were an absence of cortical waves or a decrease in their AMP. SEPs of the trigeminal nerve (V3) were recorded in 60 patients. Prolonged LAT UNIL (less frequently BIL). Much more sensitive than BAEPs: always altered when bulbar symptoms, while the MRI in no case showed syringobulbia. (They did not specify the results of these tests in the subgroup of CM1)	MEPs of the upper limbs in 60 patients. More frequent findings: PROL CCT, reduced AMP, or very polyphasic responses. (They did not specify the results of these tests in the subgroup of CM1)	―	―
Ahmmed et al. [85]	1996	T = 1 Tinnitus and mild hearing loss in the left ear	13-y-o ♀	―	―	Asymmetry in III, V LAT, and I-V IPL, more prolonged in the left ear that returned to normal values after PFD	―	―	―	―
Amoiridis et al. [121]	1996	T = 1 Dysesthesia and weakness with urinary retention	25-y-o ♂ Mild hydrocephalus	YES	YES	―	PTN SEPs: No lumbar potential (N24; Ll/iliac crest) could be obtained, whereas a high cervical potential (N33; C2/Fz) and a cortical (P40; Cz’/Fz) potential with a normal LAT were registered. MN SEPs were normal	―	AMP of the H reflex in the soleus muscle was low bilaterally, and the H reflex LAT was prolonged on the right. Motor and sensory NCS were normal. F waves in AH, EDB, or hypothenar muscles, all on the left, were not elicited	BR: Rl was absent bilaterally, and R2 LAT with left side stimulation was prolonged BIL
Kaneko et al. [120]	1996	T = 5	Mean age 18-y-o ♂	YES (cervical)	―	―	Absent or reduced cervical N13 SEP with preserved cortical responses of the upper limb were observed in 3/5 patients	MEPs LAT and AMP were normal in all patients	CMAPs LAT and AMP were normal in all patients. On the symptomatic hand, all patients showed diminished cutaneous silent periods (CSPs) up to a stimulus intensity of 15 times of sensory threshold. Diminished CSP was the only ABN finding in 2 subjects. The decrease in CSPs was more sensitive to syringomyelic changes than ABN cervical N13 SEP	―
Cristante et al. [105]	1994	T = 26 CM1 (8 with MEPs)	35–65-y-o 13♂/13♀ (Data of patients studied with MEP N/A)	YES (5)	―	―	―	Preoperative TMS MEPs: Muscles functionally impaired had their record ABN. The postoperative functional motor recovery was not reflected by improvement of the MEPs parameters	―	―
Johnson et al. [84]	1994	T = 1 Steadily progressive bilateral asymmetrical SNHL	10-y-o ♂	NO	―	CCT or I-V BIL increased: Increased I–III in one ear and III–V in the other	―	―	―	―
Strowitzki et al. [97]	1993	T = 18 CM1 = 9		YES	―	―	MN SEPs: ABN in 4 patients. PTN SEPs: ABN in 7 patients. No cortical responses were found in 6 patients and delayed responses in 2 (does not specify MN or PTN)	―	―	―
Morioka et al. [94]	1992	T = 11 (cervical SYR) CM1 = 10	24–56-y-o 3♂/7♀	YES	―	―	The most common MN SEP abnormality was the UNIL attenuation or absence of N13 with often normal N20 potentials. Spinal EPs provided information regarding abnormality responsible for the dorsal column dysfunction: the syrinx, the TE, or both	―	―	―
Nogués et al. [103]	1992	T = 13 MC1 = 7 BI = 1	19–53-y-o (m = 37.4) 7♂/6♀	T = 13	―	―	Alteration of cortical PTN and reduction or absence of cervical potential of MN	The most frequent finding was increase in CMTC	―	―
Gerard et al. [106]	1992	T = 1 BI Presenting as velar insufficiency	5-y-o ♀	―	―	―	―	―	Velar insufficiency: EMG BIL in levator palatini and anterior faucial pillars showed ample biphasic or polyphasic action potentials at rest; when the child cried, the frequency of these potentials increased poorly, and recruitment was impaired. Suspicion of denervation of the IX, X, and XI cranial nerves	―
Hendrix et al. [83]	1992	T = 3	59, 34, 64-y-o	―	―	ABN in one patient: prolonged I-V IPL on the right side. The other two patients had normal BAEPs BIL	―	―	―	―
Restuccia and Maguière [39]	1991	T = 24 MC1 = 16 (9 *)	20–74-y-o (m = 56) 10♂/14♀	T = 24	―	―	MN SEPs: ABN or absent N13 in 83% of patients with cervical SYR. Good correlation between loss of thermoalgesic sensation and absence of tendon reflexes. With associated CM: increased P14-N20 IPL	―	―	―
Jabbari et al. [93]	1990	T = 22 MC1 = 4	15–69-y-o (m = 28) 15♂/7♀	T = 22	―	―	No significant relationship between SEPs in SYR when CM coexists: in 3 of 4 patients with SYR and CM, SEPs were normal	―	―	―
Forcadas et al. [92]	1988	T = 18 MC1 = 17		12	―	―	No significant relationship between SEPs in SYR when CM coexists: in 3 of 4 patients with SYR and CM, SEPs were normal	―	―	―
Anderson et al. [91]	1986	T = 9 MC1 = 8	16–65-y-o (m = 41) 1♂/8♀	T = 9	―	―	MN SEPs: AMP reduction or absence of the cervical potential, consistent with the clinically more affected side. 7/8 cases with CM1 had a prolonged or asymmetric CCT. One case with MC-1 presented normal MN SEPs	―	―	―
Stone et al. [81]	1983	T = 1 Classified by the authors as MC2 (but without overt spinal defects), probably CM1.5	16-y-o ♂ With associated hydrocephalus	―	―	PROL I-III and I-V IPL in the left ear. Absence of wave III; I-V IPL more PROL in the right ear. Postoperative BAEPs showed BIL normalization	―	―	―	―

ABN: abnormal; AMP: amplitude; APB: abductor pollicis brevis; BAEPs: brainstem auditory evoked potentials; BI: basilar impression; BIL: bilateral; BR: blink reflex; CCT: Central conduction time; CM1: Chiari 1 malformation; CM2: Chiari 2 malformation; CMCT: central motor conduction time; CPN: common peroneal nerve; CSF: cerebrospinal fluid; CVJM: craniovertebral junction malformation; dCHEPs: dermatomal contact heat evoked potentials; dSEPs: dermatomal somatosensory evoked potentials; EMG: electromyography; FA: Fractional anisotropy; FDI: first dorsal interosseus; IH: intracranial hypotension; IPL: interpeak latency; LAT: latency; m: mean; MEPs: motor evoked potentials; MN: median nerve; MUP: motor unit potentials; NCS: nerve conduction studies; PFD: posterior fossa decompression; PROL: prolonged; PTN: posterior tibial nerve; SCOL: scoliosis; SEPs: somatosensory evoked potentials; SNHL: sensory neural hearing loss; SUR: Surgery; SYR: Syringomyelia; T: Total; TA: tibialis anterior; TE: tonsillar ectopia; TMS: transcranial magnetic stimulation; UN: ulnar nerve; UNIL: unilateral; *: Previous surgery.

## 6. Intraoperative Neurophysiological Monitoring in CM1

Most articles on this topic refer to neuromonitoring during PF surgeries. A few refer to neuromonitoring during the insertion of shunts for the treatment of syringomyelia. A third category comprises CM1 and scoliosis surgery, as well as studies reporting new techniques (Table 2).

### 6.1. Posterior Fossa Surgery

The role of intraoperative neuromonitoring (IONM) during PF surgery for CM1 is controversial, and there is still no consensus on the usefulness of the technique for the prevention of new neurological deficits nor on the most effective modality to use. Anderson et al. studied changes in intraoperative BAEPs and SEPs in 11 pediatric patients during suboccipital decompression for CM1. BAEP conduction times were compared at four surgical stages: (1) before and (2) after positioning, (3) after craniotomy, and (4) at the end of durotomy. Their data indicated that significant improvement in conduction times occurs after bony decompression and division of the atlantooccipital membrane rather than after dura opening. They also highlighted the risk of altering SEPs and BAEPs during positioning [124]. Zamel et al. reviewed the records of 80 pediatric patients, with or without syringomyelia, who underwent suboccipital decompression to treat CM1. They divided patients into two groups according to whether or not the dura mater was opened. Their data suggest that PFD with bone removal alone significantly improves BAEP conduction times in most pediatric patients with CM1 and that using duraplasty led to a further, albeit slight, improvement in conduction times in only 20% of patients [125]. Kim et al. [126] performed an alternate strategy for treating 11 symptomatic children presenting with basilar invagination associated with CM1. Their technique involved suboccipital decompression, C1 laminectomy, and duraplasty to treat CM1 and subsequent intraoperative manual reduction using accentuated cervical distraction and extension. Their results showed that immediately after reduction and fusion, SEPs improved significantly in 10 patients, while remaining unchanged from baseline in one.

In 2012, Chen et al. analyzed BAEP and SEP parameters in 13 consecutive pediatric patients who underwent suboccipital decompression to treat symptomatic CM1. They recorded the MN SEP, PTN SEP, and BAEP latencies at four stages: preoperatively, following craniotomy, following durotomy, and following closure [127]. In contrast to that reported by Anderson et al. [124] and Zamel et al. [125], they demonstrated clinically notable improvements in overall latency times in both MN SEP N20 and BAEP wave V, not only after bony decompression but also after duraplasty. Since their results showed more significant improvements in MN SEPs, they consider them the most sensitive for evaluating sufficient decompression. Incorporating an additional replacement of the bone flap retained the improvements in MN SEP and BAEPs, suggesting that this technique does not compromise the decompressive effect of the surgery.

The largest pediatric population series―with 156 patients―was reviewed in 2015 by Kennedy et al., in children with PFD but not dural opening [128]. IONM with SEPs and BAEPs was performed before and after prone positioning and during surgery. They found that 78% of patients exhibited significant BAEP conduction time improvement after bony decompression. SEPs were used to assess the lack of problems with patient positioning during surgery.

The first study evaluating the efficacy of multimodality IONM using both TcMEPs and SEPs during PFD in 21 pediatric patients was presented in 2016 by Barzilai et al. [129]. They found significant signal changes in three patients, all during patient positioning on the operating table: in two cases, the signals recovered at closure, whereas in one case, SEPs remained attenuated even after repositioning. There was no new neurological worsening after surgery. They concluded that IONM could be useful during patient positioning in PFD surgery.

In 2019, Rasul et al. also analyzed IONM data with SEPs and BAEPs at two time points—baseline before skin opening and final during skin closure—in 37 symptomatic patients who underwent PFD for CM1. They found significant SEP latency reduction in all patients. As for BAEPs, 13 patients had a reduction in their conduction time [130]. However, they could not establish a well-defined relationship between clinical outcomes and IONM changes, unlike other authors [131].

In 2020, a case report published by Krishnakumar et al. [132] evaluated the use of IONM to identify neurological safety during critical surgical steps. They reported a 13-year-old patient diagnosed with atlantooccipital dislocation with BI, hypoplastic C1 arch with CM1, and thoracic and lumbar scoliosis. Loss of TcMEPs was recorded during positioning and screw fixation, recognizing the need for a new surgical strategy. Changes in TcMEPs were reverted, and even an increase in amplitude was recorded at the end of surgery compared with baseline. During the three-month follow-up, the patient showed no new neurological deficit.

Contrary to most studies presented in the pediatric population―in which a beneficial role of IONM is suggested―the literature regarding the adult population is more skeptical as to the benefits of IONM during PFD for CM1 treatment. The results of these studies are more heterogeneous than those in pediatric patients; however, most are single case reports and, therefore, represent anecdotal evidence.

Grossauer et al. [133] published that SEPs did not significantly enhance after craniotomy or durotomy in a 32-year-old woman with CM1 and extensive cervicothoracic syringomyelia. Instead, they enhanced once the fourth ventricle opened and restored the CSF flux at the craniocervical junction.

Roser et al. [134] presented IONM data from a small series of 39 consecutive adult patients (84.6% presenting with syringomyelia) who underwent suboccipital decompression and duraplasty for treating symptomatic CM1. SEPs and TcMEPs were monitored in all patients, reporting significant changes in both SEPs and TcMEPs in only two cases related to patient positioning. They found no significant changes between initial and at-closure SEP nor TcMEP.

Heffez et al. examined the relationship between the extent of tonsillar ectopia and intraoperative changes in BAEP conduction times in an extensive series of adult CM1 cases [135]. They divided the patients into four groups depending on the position of the cerebellar tonsil. They noted a reduction of >0.1 ms in conduction times in 35–49% of patients with no statistical difference between groups or clinical outcomes. This suggests that, even with minimal tonsillar herniation, a disruption in the function of at least one pathway within the brainstem may occur and that this disturbance is improved by brainstem decompression.

In 2022, Schaefer et al. [136] suggested that CM1 suboccipital decompression surgery may be performed safely without IONM after evaluating SEPs, TcMEPs, and/or BAEPs in 93 adult patients, as they found only one instance of a transient decrease in TcMEPs, which resolved without intervention.

Some articles describe MEPs or SEPs during IONM in CM1 patients treated with other surgical techniques, like atlantoaxial stabilization surgery or endoscopic suboccipital decompression, with inconclusive results [137,138] (Table 2).

### 6.2. Surgery for the Direct Treatment of Syringomyelia in MC1

The first articles about IONM for syringomyelia in CM1 patients were published by Milhorat et al., with 32 syringomyelia patients (21 with CM1). IONM with MN SEPs demonstrated a significant decrease in N20 latency and a nonsignificant increase in N20 amplitude 30 min after syrinx decompression. They concluded that the improvement in N20 latency was indirect evidence of preexisting long tract compression. However, these conclusions are questionable in patients with CM1, in whom SEP improvements may have been caused, in part, by previous PF surgery [139,140].

Pencovich et al., in 2013, were the first to address the benefits of multimodality IONM during syringomyelia surgery. They monitored SEPs and MEPs in 13 patients with syringomyelia, six of whom also had CM1. One of the CM1 patients presented severe attenuation of left leg MEP data noted upon the midline approach to the syrinx. The catheter was subsequently removed and PFD was completed without syrinx drainage. IONM might be a valuable adjunct to spinal cord surgeries for the treatment of syringomyelia, supporting the routine use of multimodal IONM in this kind of surgery [141,142].

### 6.3. CM1 in Scoliosis Surgery

The relationship between Chiari malformation syrinx and scoliosis has been widely reported [99,123,143,144]. The associated percentage of scoliosis fluctuates from 15 to 50% in patients with Chiari malformation [145,146,147]. There is a potential risk from scoliosis surgery in patients with syringomyelia-associated scoliosis, which might be higher with longer and wider syrinxes. We found two articles with different conclusions concerning IONM during scoliosis surgery in patients with CM1. Tan et al. [148] compared radiographic findings, clinical results, and postoperative complications after posterior spinal fusion between 21 patients with CM1 and syringomyelia-associated scoliosis and 21 with idiopathic syringomyelia-associated scoliosis. As for the IONM data, TcMEPs were implemented in all patients. They noted no differences in the successful recording of baseline TcMEPs or evident TcMEP deterioration between the two groups. Patients with CM-associated scoliosis had longer syrinxes than the syringomyelia-associated scoliosis group; however, their preoperative neurological condition and IONM intraoperative findings were similar. In addition, no neurological complications were detected in either group. Shi et al. [149] studied 60 neurologically asymptomatic CM-associated scoliosis (CMS) patients (48 presented with syringomyelia) and 210 idiopathic scoliosis (IS) patients. CMS patients showed similar values of IONM compared to IS patients; however, the syringomyelia in CMS patients implied a more severe curvature and lower SEP amplitude, even though these patients had previously undergone PFD.

### 6.4. Exploratory Research on the Subject

Two articles stand out in this section. In the first, Brînzeu and Sindou carried out a research study on the functional anatomy of the accessory nerve (XI CN) via IONM (mapping) [150]. Forty-nine patients operated with dural opening in the craniocervical hinge―22 CM1 patients―participated in the study. The authors concluded that the function of each of the XI CN rootlets appears to be specific. Thus, the cranial root contributes, separately from the spinal root to the innervation of the vocal folds, which makes it a specific entity. The spinal root innervates the sternocleidomastoid and trapezius muscles with a craniocaudal motor organization of its cervical rootlets. Giampicolo et al. conducted an exploratory and preliminary study on cerebello-cortical stimulation in 10 patients undergoing PF surgery, one of them with CM1. A third of children undergoing cerebellar resections can present cerebellar mutism. According to recent evidence, it is suggested that this may arise from damage to cerebellar efferents, either uni- or bilaterally, affecting the cortex along the cerebello-dento-thalamo-cortical pathway. There is currently no neurophysiological technique available to intraoperatively monitor this pathway. This study opens an exciting research path to preserve cerebellar–cortical pathways and thus prevent cerebellar mutism in pediatric PF surgery [151].

To simplify the understanding of this extensive review, Table 3 summarizes the anatomical structures, main findings, pitfalls, and limitations of the use of each neurophysiological test in patients with CM1.

**Table 2:** Published neurophysiological studies in neuromonitoring of patients with CM1/Syringomyelia.

Author	Year	No. of Cases	Study Type	Patient Characteristics	SEP	MEP	BAEP	BR or CN Mapping	Alarm Criteria	Alerts	Findings	Conclusion
Schaefer et al. [136]	2022	93	Retrospective	17–76-y-o SYR = 53	93	92	83	―	- Decrease SEP/MEP AMP by 50% or increase SEP LAT by 10%. - BAEPs: decrease in wave V AMP by 50%, or increase in wave V LAT by 1 msec, or total loss of waves (I, III, and V)	- 1 (1.1%), which resolved spontaneously after 10 min (the patient had concomitant stenosis at C1–2)	- TE was significantly associated with unmonitorable MEPs but not with unmonitorable SEPs or BAEPs. - SYR characteristics were not significantly associated with unmonitorable MEPs, SEPs, or BAEPs. - Cerebellar symptoms were associated with unmonitorable MEPs and BAEPs but not SEPs	- PFD in CM1 may be performed safely without IONM. - In patients with additional occipitocervical pathology, IONM should be left as an option to be assessed by the surgeon on a case-by-case basis
Giampiccolo et al. [151]	2021	T = 10 CM1 = 1	An exploratory and preliminary study of cerebello-cortical stimulation	6–73-y-o 2 children	―	1	―	―	- Electrical conditioning stimuli delivered to the exposed cerebellar cortex (cStim) alone did not produce any MEP. - Paired cortico-transcortical stimulation: M1 stimulation occurred after cerebellar stimulation at fixed intervals between 8 and 24 ms. They observed significant modulation of MEPs in 8/10 patients. 5 patients showed MEP inhibition, one patient MEP facilitation, and 2 patients showed both conditions at different interstimulus intervals. - cStim preceding Tc Stim produced a significant inhibition at 8 ms (*p* < 0.0001).			- Monitoring efferent cerebellar pathways to the motor cortex is feasible in intraoperative settings. - The study has promising implications for pediatric posterior fossa surgery to preserve the cerebello-cortical pathways and thus prevent cerebellar mutism
Heffez et al. [135]	2020	488	Observational, prospective	>18-y-o Patients divided into four groups depending on the position of the cerebellar tonsils: GR 1: 0–3 mm GR 2: 3–5 mm GR 3: 5–10 mm GR 4: >10 mm Some patients presented with tethered cord in GR 4 (CM2?)	―	―	488	―	Any change of at least 0.1 msec from their initial BL	35–49% of the patients had a reduction in III–V IPL of at least 0.1 msec	Reduction in III–V IPL was observed towards completing intradural dissection or during DP, with no statistical difference between groups	- Even with minimal TE, the function of at least one pathway within the brainstem may be impaired, similar to that seen with much more extreme TE. - This impairment improves after brainstem decompression.
Krishnakumar et al. [132]	2020	1	Case report	13-y-o ♂ with atlantooccipital dislocation, BI, hypoplastic C1 arch, and CM1	1	1	―	―	N/A	- Loss of MEP in all four limbs after prone position: neck flexion was reduced by 15°, which reverted the changes in MEP. - Loss of MEP in both the upper limbs following screw tightening: C1 arch excision was made and upper limb MEPs reappeared.		- IONM can contribute to safe surgical positioning and performance. - It is essential to promptly identify and rectify any changes in neuronal, structural, and vascular integrity to help minimize neurologic sequelae.
Shi et al. [149]	2020	270	Retrospective and case-matched subgroup analysis	Scoliosis surgery 60 asymptomatic CMS patients (48 presented with SYR) vs. 210 IS patients. Case-matched patients: 60 CMS vs. 60 IS. - PFD was performed 8–12 months before correction surgery in whole patients with SYR	270	258	―	―	- Absent SEPs, UNIL or BIL prolonged LAT. - Asymmetric LAT when interside difference of LAT/AMP > 2.5 SD of normal control * LAT normalized with body height and > 2.5 SD (P37 LAT = 0.277 × height − 7.144, SD = 1.071)	- No significant difference was found between CMS and total IS patients in terms of the SEPs LAT and AMP as well as MEPs AMP. - There was no significant difference in SEPs LAT and AMP and MEPs AMP between CMS and matched IS patients. - CMS patients with SYR were correlated with lower SEPs amplitudes		- Neurologically asymptomatic CMS patients showed similar absolute values of IONM (SEPs LAT and AMP, and MEPs AMP) as compared with IS patients. - SYR in CMS patients indicated more severe curvature and a lower SEPs AMP even after PFD
Tan et al. [148]	2020	42	Retrospective and case-matched	Scoliosis surgery 21 patients with SCOL secondary to CM1 and SYR matched with 21 patients with SCOL secondary to idiopathic SYR	―	42	―	―	- Obvious MEP degeneration was defined as 40% to 80% MEP AMP loss. - Significant MEP loss was defined as >80% of MEP loss associated with high-risk surgical maneuvers	- Obvious MEP degeneration in 5 patients - Significant MEP loss in none	There were no differences in the successful recording of MEP BL	- Although patients with CM1 had longer SYR, their IONM signals during the operation were similar to those of the SCOL secondary to idiopathic SYR group. - The potential risk of SCOL surgery in patients with SYR-associated SCOL should not be ignored
Shah et al. [138]	2019	20	Observational, prospective	7–52-y-o, with CM1 that were surgically treated by atlantoaxial stabilization surgery. No bone, dural, or neural decompression	20	20	―	―	N/A	- All the patients had an immediate intraoperative improvement from their BL MEPs after the fixation was complete, ranging from 20% to 35%. - No change in the SEPs during the entire surgery in any of the patients.		The improvement in electrophysiological parameters after atlantoaxial fixation fortifies their belief that atlantoaxial instability is the cause of Chiari formation and atlantoaxial fixation is the treatment
Rasul et al. [130]	2019	37	Retrospective	<17-y-o undergoing PFD for CM1 SYR = 24 SCOL = 13	33	―	19	―	N/A	SEP: 2/33 ↑ AMP 18/33 ↓ AMP 31/33 ↓ LAT BAEP: 13/19 ↓ LAT 4/19 ↑ LAT	- SEP LAT reduced in 93.9% of patients. - >50% of patients presented SEP AMP decrease - BAEPs decreased in 68.4% of patients	- PFD for CM1 is associated with changes in SEPs and BAEPs. - A definite link between clinical outcomes and IONM was not identified
Brînzeu and Sindou [150]	2017	T = 49 22 CM1	Research study about the functional anatomy of the accessory nerve (XI CN) studied through IONM (mapping)	20–73-y-o Far-lateral decompression of tonsils at the FM in addition to posterior decompression, followed by a Y-shaped dural incision without opening the arachnoid				Rootlet and cranial root stimulation in the majority of CM1 patients				- The CN XI pair has an organization around two components: a cranial root and a spinal root with several cervical rootlets. - The cranial component contributes at least to the motor innervation of the larynx. - The spinal component largely contributes to the innervation of the sternocleidomastoid and trapezius with a precise craniocaudal myotopic organization; the rootlets destined to innervate to the sternocleidomastoid are more cranial
Kawasaki et al. [131]	2017	1	Case report	Symptomatic 7-y-o boy who underwent surgery of PFD with tonsillectomy and DP for CM1 with cervicomedullary compression + pre-SYR state at the C3-4 dorsal spinal cord	1	1	―	―	A change of 50% in AMP or a 10% change in LAT from the BL value both in MEPs and SEPs	- MEPs improved, showing increased AMP and decreased LAT after craniotomy and durotomy - SEPs improved only after durotomy		The improvement in MEPs and SEPs observed during decompression may be a good indicator for the prediction of the clinical improvement seen postoperatively
Roser et al. [134]	2016	39	Retrospective	13–65-y-o patients with CM1, undergoing suboccipital decompression and DP SYR = 33	38	33	―	―	N/A	- SEP deterioration during positioning 2/39 (↑ > 10% LAT in 4 recordings, ↓ or ↑ >50% AMP in 9 and 10 recordings) - MEP deterioration during positioning 2/39, (↓ and ↑ > 10% LAT in 11 and 10 recordings, ↓ and ↑ > 50% AMP in 14 and 17 recordings)	- No significant differences existed in the absolute BL and final LAT or AMP of MN and PTN SEPs. - There were no significant differences in the absolute BL and final LAT or AMP of APB and TA MEPs	- IONM during the primary treatment of CM1 shows only subtle non-significant changes in SEPs and MEPs without clinical correlation during suboccipital decompression
Barzilai et al. [152]	2015	22	Retrospective	21 pediatric patients aged 2–17-y-o with CM1 SYR = 18 PFD + C1 (C2/C3) laminectomy (22 surgeries)	22	22	―	―	N/A	IONM positional-related alarms in 3 patients: 1 attenuation of SEPs, 1 attenuation of MEPs, and 1 attenuation in both SEPs and MEPs.	None of the 3 patients displayed new immediate post-operative deficits	- Multimodality IONM can be helpful during patient positioning. - MEP attenuations may occur independently of SEPs. - The clinical implications of these monitoring alerts have yet to be defined
Grossauer et al. [133]	2015	1	Case report	A 32-y-o woman who underwent surgery for CM1 associated with extensive cervicothoracic SYR	1	―	―	―	A change of 50% in N20 amplitude from the BL value and a change of 10% in N20 LAT from the BL value	―	- A few minutes after opening the IV ventricle, they observed a 230% ↑ in the N20 AMP and an 8% ↓ in the N20 LAT compared to the BL value. - This SEP improvement persisted until the end of the operation	- Conduction improvement in SEPs during CM1 decompression may not always occur after bone decompression or DP. It may also happen after opening the IV ventricle and establishing CSF flow at the level of the CVJ. - Additional studies are needed to adapt the degree of decompression to each CM1 patient based on the IONM data
Kennedy et al. [128]	2015	156	Retrospective	7–20.6-y-o Suboccipital decompression without dural opening SYR = 68 SCOL = 18 m Cobb angle = 25°	156?	―	156?	―	N/A	N/A	- 78% (121) of patients exhibited at least UNIL improvement in I–V IPL after bony decompression, with a mean improvement of 0.26 ms. Once a preoperative neck position was established in the prone position with SEPs unchanged from BL, there were no adverse changes in the SEPs during any patient’s surgery	
Pencovich et al. [141]	2013	T = 13 CM1 = 6		SYR Surgery 4–61-y-o → 1 CM1 = Syrinx drainage and PFD. Level T4-T5 → 5 CM1 = Syringo-subarachnoid shunt SYR approach: → 4 midline → 2 DREZ	6	6	―	―	SEP: non-linear AMP ↓ beyond 50% or LAT ↑ of over 10%. MEP: sudden attenuation beyond 85% AMP in at least two reproducible traces after ruling out technical and anesthetic considerations	- 1 patient with absent right leg MEP BL signal. - While draining the SYR before PFD, severe attenuation of the left leg MEP data was noted upon midline approach to the SYR: the catheter was removed, and the PFD was completed without SYR drainage. New neurologic deficit: transient worsening of right hemiparesis.	- Demonstration of the reversibility of intraoperative neurological damage identified by IONM. - An immediate response resulted in rapid recovery of cord functionality	- The data collected support routine usage of IONM in SYR surgeries. - IONM can inform the surgeon of potential intraoperative threats to the functional integrity of the spinal cord, providing a helpful adjunct to spinal cord surgeries for the treatment of SYR. - More extensive prospective studies are required to show that using IONM in these operations is advantageous conclusively. - The first study to address the benefits of multimodality IONM during SYR surgery, specifically
Chen et al. [127]	2012	13		2–17-y-o Suboccipital craniotomy for symptomatic CM1. In 3, the bone flap was not replaced (craniectomy), and in 9 cases, it was (craniotomy) SYR = 3	12	―	9	―	N/A	―	- MN or PTN SEP LAT improved in all patients. - BAEPs improved in 8 patients. - No significant SEP or BAEP deterioration was seen	IONM may be used to perform suboccipital decompression in a step-by-step fashion, enlarging the craniectomy or adding additional procedures (laminectomy, DP) until positive changes are observed
Di [137]	2009	26		Endoscopic suboccipital decompression 18 months-16-y-o 0° and 30° endoscopes were adapted to perform the procedure of suboccipital craniectomy and upper cervical laminectomies SYR = 5 SCOL = 1	11	―	―	―	―	―	SEPs were monitored throughout the entire procedure for the first 11 patients, and it was then discontinued due to lack of significant benefit for the patients	SEP is still necessary, especially for the beginners of this procedure, to preclude the development of intraoperative spinal cord injury
Zamel et al. [125]	2009	80	Retrospective	2–36-y-o Group A: PFD Group B: PFD+ DP SYR = 33	80	―	80	―	- BAEP’s IPLs of I–III, III–V, and I–V waves were compared at BL, after positioning, immediately after bony decompression, and at closure. - Neurophysiologic improvement in CCT was defined as a reduction in I–V IPL at closure compared with BL	―	- A significant main interaction was found between the presence of SYR and the reduction of I–V IPL from BL to decompression. - Patients with SYR showed a significantly decreased I–V interval between BL and decompression compared with those without an SYR	- PFD with bone removal alone significantly improves conduction time in most pediatric patients with CM1. - DP allowed for further but a small improvement in conduction time in only 20% of patients, beyond that achieved by decompression alone. - None of the patients had any significant worsening of their BAEPs that would have alerted the neurosurgeon to modify the course of the surgery
Kim et al. [126]	2004	11	Retrospective	1.5–17-y-o Significant BI and CM1 They underwent a novel treatment method involving decompression, manual reduction, and posterior instrumentation-augmented fusion. SYR = 3	11	―	―	―	N/A	―	- SEPs remained stable in 10 cases and improved intraoperatively after decompression and manual reduction in 1 case. - SEPs improved significantly in 10 cases immediately after reduction and fusion. - SEPs remained unchanged from BL in 1 case	―
Anderson et al. [124]	2003	11	Observational, prospective	3–19-y-o Suboccipital decompressive procedure with DP SYR = 6	11	―	11	―	N/A	1: dramatically deteriorated of left MN SEP after turning the patient to the prone position with neck flexion. The patient was immediately repositioned in a neutral position, improving the left MN SEP.	- BAEPs: statistically significant decreased I-V IPLs after bone decompression but not after dural opening (compared to supine BL). -SEP for both sides in 10 patients remained stable throughout the procedure	- In pediatric CM1 patients, the most improvement in conduction through the brainstem occurs after bony decompression and division of the atlantooccipital membrane rather than after dural opening. - BAEPs and the SEPs indicate that these patients are at risk for neurologic injury during operative positioning with neck flexion
Milhorat et al. [140]	1997	T = 32 21 CM1		5–72-y-o In SYR with CM1: Patients with CM1 underwent suboccipital craniectomies without opening the dura + SYR shunting procedures	21	―	―	―	N/A	- 8 patients had normal SEPs, and 6 had SEP abnormalities that were unchanged 30 min after SYR decompression - 18/24 patients with pre-drainage abnormalities showed a slight but consistent N20 LAT ↓ and less consistent ↑ of N20 AMP	Significant correlation between SEP findings and SYR morphology: 6/8 patients with normal SEPs had central core cavities, and 2/8 had central cavities that extended paracentral; these types of cavities were more likely to be associated with SEP improvements after shunting (Pearson Χ^2^ = 6.47, *p* = 0.039)	- N20 LAT improvement is indirect evidence of preexisting long tract compression, whereas the decompression of perisyrinx neurons presumably caused improvements of N20 amplitudes. - These conclusions were less certain for patients with CM1 in whom SEP improvements may have been caused, in part, by decompression of the cerebellar hernia
Milhorat et al. [139]	1996	T = 13 11 CM1		12–72-y-o In SYR with CM1: PFD and SYR shunt to the PF cisterns (syringo-cisternostomy)	11	―	―	―	Continuous BL values recorded 2–3 h before SYR shunting were compared with values obtained 30 min after shunting. SEP data were analyzed by the paired *t*-test	―	30 min after SYR decompression bilateral MN SEPs demonstrated a significant ↓ in N20 LAT and nonsignificant ↑ N20 AMP	- Findings suggest that distended spinal cord cavities can produce regional ischemia, possibly reflected by SEP abnormalities and reversed by SYR decompression. - Preliminary, the IONM of SEPs can provide useful information during surgical procedures for SYR

AMP: amplitude; APB: abductor pollicis brevis; BAEPs: brainstem auditory evoked potentials; BI: basilar impression; BL: baseline; BIL: bilateral; BR: blink reflex; CCT: central conduction time; CM1: Chiari 1 malformation; CM2: Chiari 2 malformation; CMCT: central motor conduction time; CMS: Chiari malformation-associated scoliosis; CN: cranial nerve; CPN: common peroneal nerve; CSF: cerebrospinal fluid; CVJ: craniovertebral junction; dCHEPs: dermatomal contact heat evoked potentials; DP: duraplasty; dSEPs: dermatomal somatosensory evoked potentials; EMG: electromyography; FA: Fractional anisotropy; FDI: first dorsal interosseus; IONM: intraoperative monitoring; IPL: interpeak latency; IS: idiopathic scoliosis; LAT: latency; m: mean; MEPs: motor evoked potentials; MN: median nerve; MUP: motor unit potentials; NCS: nerve conduction studies; PF: posterior fossa; PFD: posterior fossa decompression; PROL: prolonged; PTN: posterior tibial nerve; SCOL: scoliosis; SEPs: somatosensory evoked potentials; SNHL: sensory neural hearing loss; SYR: Syringomyelia; T: Total; TA: tibialis anterior; TcMEP: transcranial-electric motor evoked potential; TE: tonsillar ectopia; TMS: transcranial magnetic stimulation; UNIL: unilateral; *: Previous surgery; ↑: increase; ↓: decrease.

## 7. Conclusions

Many published articles deal with the techniques performed in the neurophysiology laboratory in CM1 patients; however, most are of limited usefulness as they focus on heterogeneous, non-systematized series or case reports, where only the most affected patients (most with syringomyelia) are investigated. Most studies are about SEPs, probably due to the frequent association of CM1 with syringomyelia. The most common SEP alteration (both MN and PTN) was increased CCT, occasionally associated with altering the cervical potential. We did not find any publication regarding LEPs and only one about CHEPs and CM1 with syringomyelia, although, as we mentioned previously, the sensibility of these tests is superior to classic SEPs in detecting pure spinothalamic lesions. BAEPs are the second most frequently studied modality in the literature, with the most common finding being an increased I–V interval, suggesting central or retrocochlear involvement. MEPs are the third most frequently studied parameter, and the most commonly observed finding is an increased CMCT. Regarding EMG, most results indicate major or minor denervation, confirming the presence of an anterior horn cell lesion when patients have syringomyelia.

Our view is that the utility of neurophysiological studies in patients with CM1 depends on the clinical context. Thus, when there is apparent symptomatology, our results suggest that neurophysiological explorations do not add clinically relevant information, nor are they useful in establishing which patients should undergo surgical treatment. Hence, it is not necessary to include them in the routine study. Nevertheless, the fact that half of our patients in whom MC1 was discovered incidentally had abnormal evoked potentials, establishing objective evidence of subclinical dysfunction. Therefore, this is helpful in the follow-up of patients who have apparently become stable and who require demonstrating subclinical progression.

Concerning PF surgery IONM, the published studies we found were also heterogeneous series, not only as regards patient characteristics (such as different age ranges, with or without syringomyelia, and CVJ malformations) but also the type of neurophysiologic modality used for monitoring. Most authors used SEPs, with BAEPs or MEPs having been used less frequently. Surprisingly, we did not find any publication on the control of cranial nerves by reflexes (such as BR or LAR), Co-MEP, or by evaluating free-EMG activity. Since the lower cranial nerves can be affected by this pathology and may be at risk in certain patients in whom tonsillar coagulation or resection are required or in patients at high risk from surgery, such as patients with CM1.5, with the currently available equipment, when intraoperative monitoring is decided, the goal is multimodal IONM including monitoring of the lower cranial nerves. Some authors have proposed IONM during surgery in patients with CM1 in the following three scenarios: (1) during patient positioning before surgery, (2) to determine when adequate decompression has been performed to plan surgery, and (3) to notice intraoperative neurophysiological worsening when intradural manipulation is planned. For surgical positioning in patients with CM1 (scenario 1), the rationale for IONM is that it can reduce the risk of neurological injury while it can still be reversed, particularly in patients with CM1.5 in whom retrocurved odontoid and partial anterior compression of the cervico-medullary junction are present. In scenario 2, the most debatable feature is using IONM to limit the degree of PFD and decide whether to open the dura. Analyzing some series that compare MRI studies of CM1 pediatric patients treated with an extradural procedure and others undergoing duraplasty, the normalization rate of tonsillar descent is seen to be clearly higher in the group of patients treated with duraplasty [125,152,153]. Considering these findings, we cannot believe the best surgical option can be decided based on the IONM data. Regarding scenario 3, none of the reports reviewed concerning CM1―without associated complex CVJ malformations―presented any significant IONM alarm. These findings reinforce that the frequency of new neurological deficits in the PFD surgery of CM1 patients without complex CVJ malformations is very low once the patient has been positioned.

In conclusion, the evidence suggests that the only advantage of IONM in CM1 is during patient positioning and only in specific patients (CM1.5), making the regular practice of IONM challenging to justify, at least with the IONM modalities studied so far.

## Figures and Tables

**Table 3:** Summary of the characteristics of neurophysiological tests in CM1.

Neurophysiological Tests	Pathway	Main Findings	Pitfalls and Limitations
SEP	Dorsal columns or lemniscal system	Increased N13-N20 interval. Reduced or absent cervical potential (N13)	- Associated neuropathy, other forms of cervical spinal stenosis, and cervical myelopathy - Requires patient collaboration (especially relaxation)
BAEP	Auditory from cochlea to superior pons	Increased I–V interval, suggesting central or retrocochlear involvement	- Associated hypoacusia of cochlear origin - Requires patient collaboration (especially relaxation)
MEP	Pyramidal system or corticospinal tract	Increased CMCT	- Variability related to maturative age - Requires patient collaboration
EMG/NCS	Peripheral nervous system (roots/anterior horn, nerves and muscles)	Preganglionic pattern at NCS (preserved sensory neurography) with acute or chronic denervation at EMG, compatible with the suspicion of anterior horn cell lesion if syringomyelia is present	- Concomitant neuropathy or radiculopathy secondary to spondylosis
Brainstem reflexes (Blink reflex)	Involved cranial nerves, nucleus, and pathways in the brainstem	Altered R1 and/or R2	- Variability related to maturative age - Repetitive high frequencies stimuli cause adaptation phenomenon - Lack of published follow-up studies for CM1 and other brainstem reflexes
Intraoperative neurophysiological monitoring	All of the pathways described above	Evidence for benefit during positioning	Anesthetic considerations

BAEP: brainstem auditory evoked potential; CMCT: central motor conduction time; EMG: electromyography; MEP: motor evoked potential; NCS: nerve conduction studies; R: blink reflex response; SEP: somatosensory evoked potential.
